# Personalized Approaches to Hypothyroidism: The Role of Triiodothyronine (T3) in Thyroid Hormone Replacement

**DOI:** 10.7759/cureus.90685

**Published:** 2025-08-21

**Authors:** Angela D Mazza

**Affiliations:** 1 Endocrinology, Metabolic Center for Wellness, Oviedo, USA

**Keywords:** combination thyroid therapy, deiodinase polymorphism, hypothyroidism, levothyroxine (t4), personalized medicine in endocrinology, triiodothyronine (t3)

## Abstract

Levothyroxine (T4) monotherapy remains the standard of care for hypothyroidism worldwide. However, a subset of patients continues to report persistent symptoms, such as fatigue, depression, and cognitive difficulties, despite normalized thyroid-stimulating hormone (TSH) levels. This has reignited clinical interest in the active thyroid hormone triiodothyronine (T3) and combination T4/T3 therapies. This review explores the physiological distinctions between T4 and T3, emphasizing the importance of peripheral T4-to-T3 conversion and the role of deiodinase polymorphisms (e.g., DIO2 Thr92Ala) that may impair this process in certain individuals. The evidence for T3-containing therapies is critically evaluated, including synthetic liothyronine and desiccated thyroid extract, comparing their pharmacokinetics, clinical outcomes, and safety profiles with T4 monotherapy. Emerging data suggest that some patients may benefit from combination therapy, particularly in symptom resolution and quality-of-life measures, although robust long-term outcomes remain under debate. Current clinical guidelines from major thyroid societies continue to endorse T4 monotherapy, citing inconsistent trial results and potential cardiovascular risks associated with T3. Nonetheless, a growing movement toward personalized medicine, including genetic profiling, patient-reported outcomes, and individualized dosing, may pave the way for more nuanced treatment strategies. This review underscores the need for a balanced, patient-centered approach to thyroid hormone replacement, integrating evolving science with clinical experience to optimize care for individuals with hypothyroidism.

## Introduction and background

“If my thyroid levels are normal, why don’t I feel that way?” This question is increasingly echoed by patients diagnosed with hypothyroidism who, despite achieving target thyroid-stimulating hormone (TSH) levels on levothyroxine (LT4) monotherapy, continue to experience fatigue, weight gain, cognitive slowing, and other symptoms. Thyroid hormones play a central role in regulating metabolic homeostasis, neurocognitive function, cardiovascular health, and cellular energy production [1]. The thyroid gland primarily secretes thyroxine (T4), a prohormone that must be converted in peripheral tissues into triiodothyronine (T3) - the active form that binds nuclear receptors to influence gene expression and mitochondrial activity [2,3]. While LT4 monotherapy has long been the standard of care due to its ability to normalize serum TSH [4], emerging evidence suggests that TSH alone may not reflect tissue-level euthyroidism for all individuals. Inadequate T4-to-T3 conversion, genetic polymorphisms, and individual variations in deiodinase activity may contribute to persistent symptoms [5,6]. These challenges have sparked renewed interest in the role of T3-based and combination therapies as part of a more personalized approach to thyroid hormone replacement [7,8].

## Review

Does thyroid hormone physiology and conversion happen in real life? A gap between guidelines and practice

Despite the official guidelines, real-world treatment doesn’t always match the textbook. Surveys show that many clinicians, especially in Europe and North America, do offer T3 or combination therapy for patients who still have symptoms despite “normal” TSH levels and optimal T4.

Thyroid hormones are essential for regulating metabolism, growth, and development, and the body works hard to keep them in balance. Most of what the thyroid gland releases is T4, a relatively inactive hormone that must be converted into the more active form, T3, to do its job in cells. This conversion happens in various tissues throughout the body and is controlled by a group of enzymes called deiodinases: DIO1, DIO2, and DIO3 [1,2]. DIO1 and DIO2 help activate T4 by turning it into T3, while DIO3 does the opposite. DIO3 turns T4 and T3 into inactive forms like reverse T3 (rT3), essentially dialing down thyroid hormone activity [9]. These enzymes are not evenly distributed. For instance, DIO2 is found in high levels in the brain and brown fat, helping ensure those tissues get enough active T3 even if blood levels are low [10,11].

Why does this matter? Because thyroid hormone action is not just about what is in the bloodstream. T3 binds far more effectively than T4 to receptors in key organs like the heart, liver, and brain, triggering changes in gene expression and cellular energy use [12]. This means that even if lab tests show “normal” thyroid hormone levels, someone may still experience symptoms if their tissues are not converting or responding to those hormones properly. This is a critical consideration for truly understanding and treating thyroid disorders.

Conventional Therapy: Levothyroxine (T4)

LT4 has been the main treatment for hypothyroidism since the 1970s. It is favored because it has a long half-life, is easy to dose, and reliably brings TSH levels back to normal [4]. LT4 is a synthetic version of T4, which the body normally converts into the active hormone T3 in tissues throughout the body [2]. Studies have shown that LT4 works well for many people, especially those with hypothyroidism from Hashimoto’s thyroiditis or thyroid surgery [13].

However, even when TSH levels are in the target range, about 10-15% of patients continue to experience symptoms like fatigue, brain fog, weight gain, and low mood [14,15]. These ongoing issues may be due to problems with converting T4 to T3, differences in how individuals process thyroid hormones, or reduced sensitivity to T3 at the cellular level [6]. Because of this, many experts are questioning whether normal TSH levels alone are enough to judge treatment success and are advocating for more personalized approaches that also consider how patients feel, not just what the lab results show [5].

T3 Therapy: Mechanistic and Clinical Rationale

T3 is the active form of thyroid hormone and has powerful effects throughout the body. It works by turning on genes inside cells through thyroid hormone receptors, especially the thyroid hormone receptor beta (TRβ), which it binds to most strongly. T3 boosts energy production by increasing the number and activity of mitochondria, which helps the body make more adenosine triphosphate (ATP), or energy, and stay warm through thermogenesis [16,17]. It is also essential for brain function, supporting memory, mood, and overall cognitive health by helping regulate neurotransmitters and synaptic activity [18].

Some people on LT4 therapy continue to feel tired, depressed, or mentally foggy even when their TSH and free T4 (FT4) levels look normal. One reason may be that their tissues are not getting enough active T3. This has led to growing interest in adding or switching to liothyronine (LT3), especially for those who may not convert T4 to T3 efficiently [7].

Synthetic T3 acts differently in the body compared to LT4. It is absorbed more quickly, has a shorter half-life of about 24 hours, and “kicks in” faster, which can offer quicker symptom relief for some patients [19]. But because of its short duration, it can cause fluctuations in T3 levels throughout the day, which may increase the risk of side effects like heart palpitations or mood swings if not carefully managed [20].

Certain genetic variations, like the Thr92Ala change in the DIO2 gene, can affect how well someone converts T4 into T3. People with this gene variant may have lower levels of T3 inside their cells and may not feel well on T4 alone [6]. Some studies suggest they might benefit from a combination of T4 and T3 therapy, although results have been mixed and more research is needed [8].

As we move toward more personalized medicine, considering a person’s genetics, lab results, and symptoms together may help doctors better identify who could benefit most from adding T3 to their treatment plan.

Combination Therapy: Promise and Pitfalls

For patients who still feel unwell on LT4 alone, even when their TSH levels are “normal," adding LT3 has become an area of interest. Over the past 20 years, researchers have conducted several randomized controlled trials (RCTs) and meta-analyses to compare T4-only treatment with combination T4 + T3 therapy. While most of these studies haven’t shown major advantages in lab results or hormone levels, some have found that a portion of patients report feeling better - experiencing improved energy, mood, or mental clarity [2,21].

A widely cited 2006 meta-analysis by Grozinsky-Glasberg and colleagues concluded that combination therapy did not significantly improve mood, cognition, or quality of life overall [22]. However, that analysis and many earlier trials may not have captured important differences in how individuals respond to treatment. For example, more recent studies suggest that people with certain genetic traits, like the Thr92Ala variant in the DIO2 gene (which affects T4-to-T3 conversion), might benefit more from combination therapy [23-25]. Still, many of these studies are underpowered for subgroup analysis or use inconsistent symptom scales, making it difficult to draw firm conclusions.

Dessicated thyroid extract (DTE)

DTE is a natural thyroid hormone replacement derived from the dried thyroid glands of pigs (porcine origin). It contains both T4 and T3, along with other iodinated compounds that may reflect the natural complexity of thyroid output [4,5]. DTE products, such as Armour® Thyroid and NP Thyroid®, offer a fixed T4:T3 ratio of about 4:1, which is higher in T3 than the human thyroid’s typical 14:1 ratio [26]. That being said, it is important to note that when looking at the thyroid’s actual physiologic production compared to a once-daily dosing of a tablet, this is not an accurate comparison. While this formulation may benefit some individuals, particularly those with impaired T4-to-T3 conversion or persistent symptoms on T4 alone, it can lead to variable T3 levels and symptoms such as palpitations, anxiety, or sleep disturbance in others when the dose is inappropriate.

A common misconception is that DTE is unregulated or inconsistent in quality. In fact, commercially available DTE products in the United States are FDA-regulated as prescription drugs. The manufacturing of these products is subject to current Good Manufacturing Practices (cGMP), and thyroid hormone content is monitored for consistency and potency to meet United States Pharmacopeia (USP) standards [27]. However, while DTE is regulated, it is important to note that many of these formulations were marketed prior to modern FDA drug approval processes and have not undergone full contemporary approval under a New Drug Application (NDA).

Though some small studies and patient surveys suggest that DTE may improve quality of life for certain individuals compared to T4 alone, these findings are limited by small sample sizes, lack of placebo controls, short follow-up periods, and inconsistent methodologies. Larger, well-designed clinical trials are needed to clarify the safety, efficacy, and ideal patient populations for DTE.

Compounded Preparations

Compounded thyroid hormone preparations, including customized formulations of LT4, LT3, and DTE, offer a tailored approach for patients who do not respond optimally to commercially available therapies. These preparations can be adjusted to specific T4:T3 ratios, doses, or delivery systems (e.g., sustained-release T3 (SR-T3)), potentially improving symptom control in individuals with persistent fatigue, cognitive dysfunction, or mood disturbances despite normalized TSH levels. Benefits include personalization for patients with sensitivities to excipients or unique dosing needs. However, drawbacks include limited large-scale evidence of efficacy and safety, lack of FDA approval for compounded formulations, and potential variability in potency and bioavailability between compounding pharmacies due to differences in sourcing, quality control, and adherence to USP standards [28,29]. Notably, studies have found that compounded thyroid products may exhibit inconsistent hormone content compared to FDA-approved preparations, raising concerns about stability and therapeutic reliability [30]. Therefore, while compounded options serve a niche role in individualized care, they require careful prescribing and monitoring.

Patient experience and practical challenges

More attention is now being paid to patient-reported symptoms, like fatigue, depression, and brain fog, because up to 15% of people taking T4 alone continue to struggle with these issues, even when their thyroid labs look fine [5]. In some studies, patients on combination therapy report feeling better subjectively, even when their TSH or FT4 levels don’t change [6]. For example, in a well-known trial by Appelhof et al., some participants preferred combination therapy despite no measurable improvements on formal mood or memory tests [7]. This raises an important question: should symptom improvement alone be enough to guide therapy, even if the labs say otherwise?

That said, combination therapy comes with real challenges. T3 has a short half-life, acts quickly, and can cause hormone levels to spike shortly after taking a dose. This can lead to symptoms like palpitations, anxiety, or energy crashes if not dosed carefully [8]. There’s also no consensus on the ideal T4-to-T3 ratio, and slow-release T3 formulations aren’t widely available, making it harder to fine-tune treatment [9]. These practical limitations, alongside mixed clinical evidence, make combination therapy a promising but still controversial option. More high-quality, personalized research is needed to determine who benefits most and how best to deliver that care.

Toward personalized thyroid care

Personalized medicine is changing the way we approach thyroid treatment. Instead of relying on standard doses based on population averages, this evolving model focuses on tailoring therapy to each person’s biology and symptoms. One exciting area of research looks at genetic differences that affect how thyroid hormones are processed and used in the body. For example, a common genetic variant called Thr92Ala in the DIO2 gene, which is responsible for converting T4 into the active T3 hormone inside cells and has been linked to reduced enzyme activity. This means people with this variant might produce less T3 where it matters most, even if their blood tests look normal while taking LT4 alone [6,31].

Other genetic differences, like those in the SLCO1C1 and MCT10 genes, may affect how thyroid hormones enter cells. These variations could further explain why some people don’t feel well on standard treatment, despite having “normal” lab results [32]. Together, these findings support the idea of genotype-guided therapy - using a person’s genetic profile to help decide whether adding LT3 might improve their symptoms.

Listening to the patient: beyond lab results

Alongside genetics, patient-reported symptoms are becoming an important part of thyroid care. Tools like the ThyPRO questionnaire help track issues such as fatigue, brain fog, emotional well-being, and overall quality of life [33]. These tools can capture lingering symptoms that blood tests might miss, giving doctors a clearer picture of who might benefit from a change in treatment [34].

Using a customized T4:T3 ratio is one example of this individualized approach. Instead of giving everyone the same fixed dose of LT4, doctors can adjust the balance between LT4 and LT3 based on lab values, symptoms, and possibly genetics. While the best ratio is still debated, some studies suggest that mimicking the body’s natural thyroid output of about a 13:1 to 16:1 T4:T3 ratio may work better for some people than LT4 alone [20].

This approach reflects a broader shift toward precision endocrinology, which aims not just to normalize lab numbers but to help patients truly feel better and regain their quality of life.

New frontiers in thyroid therapy

As more people continue to struggle with symptoms despite taking LT4, researchers are exploring new ways to improve thyroid hormone treatment (Table 1). One major development is SR-T3. Unlike the standard fast-acting T3, which can cause sharp spikes in hormone levels, sometimes leading to heart palpitations or mood swings, SR-T3 is designed to release slowly throughout the day. This better mimics how the body naturally produces T3, potentially making it a safer and more stable option [35].

**Table 1:** Future thyroid formulations and directives TRβ: thyroid hormone receptor beta; T3: triiodothyronine; T4: levothyroxine; NASH: nonalcoholic steatohepatitis; TRα: thyroid hormone receptor alpha; FDA: U.S. Food and Drug Administration

Therapy/Agent	Mechanism/Description	Potential Benefits	Limitations/Status
Sustained-release T3 (SR-T3)	Modified-release liothyronine designed to mimic the circadian T3 rhythm and reduce serum peaks	More stable T3 levels, fewer cardiac/mood side effects, closer to physiologic secretion	Limited availability; not yet FDA-approved; long-term data pending
TRβ-selective analogs (e.g., eprotirome, VK2809)	Synthetic agents selectively targeting TRβ to modulate liver/lipid metabolism	Lipid-lowering, liver fat reduction, and potential NASH application with fewer TRα-mediated side effects	Not approved for hypothyroidism; still in trials for metabolic diseases
Bioidentical compounded T3/T4	Custom-compounded formulations tailored to patient-specific T4:T3 needs, including slow-release options	Personalized dosing, improved symptom control in T4 non-responders	Variable compounding standards; lack of large-scale clinical data
Future directions (e.g., nanoparticle, transdermal delivery)	Experimental delivery systems aiming for tissue-specific targeting and improved absorption	Precision hormone delivery with minimal systemic peaks	Experimental stage; delivery mechanisms under development

Early studies show that SR-T3 helps maintain steadier T3 levels and causes fewer side effects like rapid heartbeat [36]. However, SR-T3 is still experimental - it isn’t widely available, and it hasn’t been approved by the FDA yet. More research is needed to understand how effective and safe it is over the long term.

Targeting the right tissues: TRβ-selective analogs

Another exciting area of research involves thyroid hormone analogs - medications that act like thyroid hormones but are designed to target specific tissues. Some of the most promising compounds, like sobetirome and VK2809, are TRβ-selective agonists. These drugs are built to activate the thyroid hormone receptors in the liver, TRβ, where they help lower cholesterol and reduce liver fat, while avoiding activation of the receptors in the heart and bones, TRα, where side effects can occur [37,38].

In clinical trials, these analogs have shown significant promise - lowering low-density lipoprotein (LDL) cholesterol and improving liver health in conditions like high cholesterol and metabolic dysfunction-associated steatotic liver disease (MASLD) [39]. Although they aren’t used to treat hypothyroidism yet, they offer a glimpse into the future of more targeted, tissue-specific thyroid treatments.

Looking ahead: precision and personalization

The future of thyroid care is heading toward more personalized, bioidentical, and targeted treatments. Custom-compounded formulations of T3 and T4, often created by specialty pharmacies, are already helping patients who need unique dosing schedules or slow-release options [40]. Experimental delivery methods, like nanoparticles or transdermal patches, are also being explored.

As we learn more about how genetics (like DIO2, MCT8, or THRB variants) affect individual hormone metabolism and tissue response, thyroid treatment is moving into the realm of precision medicine. This means using a combination of genetics, biomarkers, and patient-reported symptoms to guide therapy, not just relying on blood tests alone [2]. These innovations aim to close the gap between “normal labs” and actually feeling well - a goal shared by both patients and providers.

T3 therapy: why it needs careful oversight

Using LT3 as part of thyroid hormone therapy, whether alone or in combination with LT4, warrants careful clinical oversight due to its rapid onset of action, shorter half-life, and higher potency. Historically, concerns have centered on its potential cardiovascular effects, particularly in older adults or those with preexisting heart disease [4,41]. Supraphysiologic doses of T3 may transiently increase heart rate, enhance myocardial oxygen demand, or predispose to arrhythmias such as atrial fibrillation. However, several studies have not consistently demonstrated a significantly increased risk of adverse cardiovascular outcomes when T3 is used at physiologic or low doses, particularly in combination regimens. For example, a systematic review by Grozinsky-Glasberg et al. (2006) found no clear increase in adverse events in patients treated with combination T4/T3 therapy compared to T4 monotherapy. Similarly, an RCT by Escobar-Morreale et al. (2005) reported no significant changes in cardiac function or arrhythmia risk among women receiving T3-inclusive therapy. Nevertheless, transient peaks in serum T3 after immediate-release LT3 dosing can occur, emphasizing the need for individualized dosing strategies and close monitoring, especially in those with cardiovascular comorbidities.

Other health effects: bones, mood, and metabolism

T3 therapy doesn’t just affect the heart. It can also impact bones, mood, and metabolism. Too much thyroid hormone speeds up bone turnover, which can increase the risk of osteoporosis and fractures, especially in postmenopausal women [42]. However, when combination therapy is properly dosed, it has not been consistently shown to cause more bone loss than T4 alone [8].

Some patients report improved mood, energy, and mental clarity when T3 is added to their treatment, especially if they still have symptoms like fatigue or brain fog despite normal TSH levels on T4-only therapy [7]. T3 also boosts metabolism by increasing mitochondrial activity and heat production, which may help with weight control and cholesterol levels, but these effects can go too far if the dose is too high, leading to unwanted energy loss or metabolic stress [5].

Monitoring: more than just a TSH number

Keeping patients safe and well on thyroid therapy means looking beyond just the TSH lab result. While TSH has traditionally been the main marker used to assess thyroid function, it doesn’t always reflect what’s happening inside cells. This holds even more true in people taking T3 or combination therapy [5]. A more complete picture comes from also checking FT4, free T3 (FT3), and sometimes rT3.

RT3 can help identify problems with hormone conversion or the effects of stress on thyroid function. Tracking both FT3 and FT4 helps assess the balance between the two hormones, which is especially important in combination therapy [2]. Just as important as labs are the patient’s own experiences - tools like the ThyPRO questionnaire can capture lingering symptoms that labs might miss.

In the end, the goal of thyroid treatment isn’t just to get “normal” lab values, but to help people feel better, regain their energy, and improve their quality of life.

Ongoing debate: should T3 be part of hypothyroidism treatment?

The treatment of hypothyroidism continues to be a hot topic, especially when it comes to the role of T3 and combination therapy. Most major medical organizations, including the American Thyroid Association (ATA), European Thyroid Association (ETA), and British Thyroid Association (BTA), recommend LT4 alone as the standard treatment for most people with hypothyroidism. Their support for T4 monotherapy is based on its long half-life, steady blood levels, and ability to reliably bring TSH back to normal - the marker they consider most accurate for tracking thyroid status [20,22].

While these guidelines do acknowledge that some patients continue to feel unwell on T4 alone, they don’t recommend routine use of T4/T3 combination therapy as first-line therapy. The main concerns are inconsistent results in clinical trials and the possible risks of T3, such as heart palpitations or overactive thyroid symptoms, particularly in older adults or people with heart conditions. 

What happens in real life? A gap between guidelines and practice

Despite the official guidelines, real-world treatment doesn’t always match the textbook. Surveys show that many clinicians, especially in Europe and North America, do offer T3 or combination therapy for patients who still have symptoms despite “normal” TSH levels and optimal T4 dosing [4,5,42,43]. Many patients are also exploring other treatment options, often driven by frustration with ongoing symptoms and encouragement from patient advocacy groups.

This disconnect has fueled growing interest in personalized thyroid hormone replacement. Recent studies and consensus documents highlight the role of genetic polymorphisms (e.g., DIO2 Thr92Ala, MCT10 variants), variable deiodinase activity, and patient-reported outcomes in shaping individual responses to therapy [6,24,44,45]. Trials and observational studies suggest that select subgroups of patients may experience symptomatic benefit from individualized regimens, supporting the call for more targeted research in this area [4,46,47]. This evolving emphasis on personalization underscores the need for more robust research to define which subgroups of patients are most likely to benefit from individualized regimens to provide an evidence base for clinical decision-making.

Conventional versus functional medicine: different philosophies

The divide between mainstream and functional approaches to thyroid care reflects two different philosophies. Conventional endocrinology focuses heavily on lab results, especially TSH and standardized treatments, which are based on large population studies. Functional and integrative practitioners, by contrast, emphasize personalized care. They often consider a wider range of factors like FT4, FT3, rT3, adrenal function, and nutrient status, along with a strong focus on how the patient feels [48].

Critics argue that this broader approach lacks strong clinical trial evidence and may risk overtreatment. But supporters believe it addresses real-world gaps, particularly for patients whose symptoms persist despite having “normal” labs. As science continues to uncover how genetics, tissue-level hormone activity, and cellular transport affect thyroid health, there’s growing agreement that one-size-fits-all treatment may not work for everyone. This opens the door for future guidelines to combine lab-based targets with symptom-based care for a more balanced, individualized approach.

Moving toward personalized thyroid care

As we continue to learn more about how thyroid hormones act in different tissues, how genetic differences affect metabolism, and how symptoms relate to lab results, the future of thyroid care is moving toward more personalized, patient-centered strategies. However, current evidence from RCTs and meta-analyses has been mixed. While some trials suggest that combination T4/T3 therapy or desiccated thyroid extract may improve patient-reported outcomes such as fatigue, mood, or quality of life, other studies show no significant difference compared with T4 alone [22,23]. Meta-analyses have reinforced these inconsistencies, often concluding that the benefits of combination therapy remain unproven for the general hypothyroid population [15,22].

A critical limitation of the studies that we have is their short duration, relatively small sample sizes, and reliance on heterogeneous outcome measures, which may obscure benefits in subgroups more likely to respond (e.g., those with deiodinase polymorphisms). Furthermore, most RCTs were not designed to capture long-term safety endpoints such as cardiovascular or skeletal outcomes. These methodological gaps underscore why, despite guideline recommendations for T4 monotherapy, the discussion around more individualized approaches continues. Personalized strategies, therefore, remain an emerging but not yet fully validated direction, requiring larger and better-targeted studies before they can be broadly adopted.

Key takeaways

T4 monotherapy remains the standard of care, effectively normalizing TSH in most patients, but up to 15% continue to experience persistent symptoms such as fatigue, depression, and cognitive decline despite biochemical euthyroidism. T3 therapy, especially in combination with T4, may offer symptomatic relief in select patients, particularly those with impaired peripheral conversion, genetic polymorphisms (e.g., DIO2 Thr92Ala), or poor quality of life on T4 alone. Clinical guidelines (ATA, ETA, BTA) remain conservative in endorsing T3 use due to limited and inconsistent trial data, but real-world practice increasingly reflects personalized approaches in symptomatic patients. Emerging therapies, including SR-T3 and TRβ-selective analogs, aim to improve safety, mimic physiologic hormone rhythms, and offer targeted metabolic benefits while minimizing systemic risks (Table 1). A more personalized, symptom-informed treatment model - integrating genetic factors, patient-reported outcomes (e.g., ThyPRO), and broader hormone monitoring (TSH, FT3, FT4, rT3) - is critical for optimizing thyroid care in patients with unresolved symptoms.

## Conclusions

While LT4 remains the standard and guideline-recommended treatment for hypothyroidism, it doesn’t work equally well for everyone. More and more evidence shows that some patients continue to experience symptoms, like fatigue, low mood, and brain fog, even when their TSH levels are back to normal. For these individuals, adding LT3, changing to DTE, or using newer T3 formulations may offer additional benefits. This is especially true for people who have trouble converting T4 into active T3, or who carry certain genetic variations like the DIO2 Thr92Ala polymorphism, which may limit how well their cells use thyroid hormone. Research and real-world experiences show that normal lab values don’t always mean patients feel well, which highlights the need for a more individualized, symptom-based approach to treatment.

As we continue to learn more about how thyroid hormones work in different tissues, how genetic differences affect metabolism, and how symptoms relate to lab results, the future of thyroid care is moving toward personalized, patient-centered strategies. These approaches aim not only to balance lab values but also to improve how people actually feel, helping more patients achieve true wellness, not just biochemical “normal.”
